# HMGB1 Localization during Experimental Periodontitis

**DOI:** 10.1155/2014/816320

**Published:** 2014-02-20

**Authors:** Andressa Vilas Boas Nogueira, João Antonio Chaves de Souza, Rafael Scaf de Molon, Elyne da Silva Mariano Pereira, Sabrina Garcia de Aquino, William V. Giannobile, Joni Augusto Cirelli

**Affiliations:** ^1^Department of Diagnosis and Surgery, School of Dentistry at Araraquara, Universidade Estadual Paulista (UNESP), Rua Humaitá, 1680, 2° Andar, Centro, 14801-903 Araraquara, SP, Brazil; ^2^Department of Periodontics and Oral Medicine, School of Dentistry, University of Michigan, 1011 N. University Avenue, Ann Arbor, MI 48109-1078, USA

## Abstract

*Aim.* This study sought to investigate the *in vitro* expression profile of high mobility group box 1 (HMGB1) in murine periodontal ligament fibroblasts (mPDL) stimulated with LPS or IL-1**β** and *in vivo* during ligature- or LPS-induced periodontitis in rats. *Material and Methods.* For the *in vivo* study, 36 rats were divided into experimental and control groups, and biopsies were harvested at 7–30 d following disease induction. Bone loss and inflammation were evaluated. HMGB1 expression was assessed by immunohistochemistry, qPCR, and Western blot. *Results.* Significant increases in mPDL HMGB1 mRNA occurred at 4, 8, and 12 h with protein expression elevated by 24 h. HMGB1 mRNA expression in gingival tissues was significantly increased at 15 d in the LPS-PD model and at 7 and 15 d in the ligature model. Immunohistochemical staining revealed a significant increase in the number of HMGB1-positive cells during the experimental periods. *Conclusion.* The results show that PDL cells produce HMGB1, which is increased and secreted extracellularly after inflammatory stimuli. In conclusion, this study demonstrates that HMGB1 may be associated with the onset and progression of periodontitis, suggesting that further studies should investigate the potential role of HMGB1 on periodontal tissue destruction.

## 1. Introduction

High mobility group box 1 (HMGB1) is a nonhistone DNA-binding nuclear protein present in almost all eukaryotic cells. Among other functions in the nucleus, HMGB1 promotes DNA bending, regulates DNA transcription, and participates in DNA repair. HMGB1 can be released to the extracellular space acting like a proinflammatory cytokine [1, 2]. This secretion occurs mainly after infection or necrosis/apoptosis, respectively, by an active or a passive mechanism. Macrophages and other inflammatory cells can release HMGB1 after stimulation with LPS or cytokines, and in turn HMGB1 increases cytokine production [3]. Indeed, HMGB1 increases the number of adhesion molecules on endothelial cells, activates dendritic and T cells, and stimulates the differentiation of osteoclast precursor in the presence of RANKL [4–7]. Extracellular HMGB1 also has the ability to form complexes with agents that induce inflammation such as LPS, IL-1*β*, and other danger signal molecules. These complexes increase cytokine production as shown in experimental models of systemic lupus erythematosus, endotoxemia, rheumatoid arthritis, and osteoarthritis [1, 8–10].

Extracellular HMGB1 mediates an inflammatory response by binding to receptor for advanced-glycation end products (RAGE) and toll-like receptor- (TLR-) 2, toll-like receptor-4, and toll-like receptor-9, [11, 12] which are implicated in inflammatory processes. As a result, HMGB1 plays a role in many acute and chronic inflammatory conditions, such as rheumatoid arthritis, diabetes mellitus, atherosclerosis, lupus, cancer, and periodontal disease [10, 13–18]. In addition, many attempts have been made to block or neutralize HMGB1 using antibodies and antagonist proteins or release inhibitors in models of inflammatory diseases. This action has shown beneficial effects with suppression of the inflammatory response, suggesting that HMGB1 could be considered as an inflammatory mediator and a therapeutic target [3, 19, 20].

In patients with periodontal disease, higher HMGB1 concentration and number of positive cells have been found, respectively, in gingival crevicular fluid and inflamed gingival epithelial cells compared to healthy control sites [21]. Also, this protein is upregulated in human gingival fibroblasts after stimulation with LPS from periodontal pathogens, in human gingival epithelial cells and fibroblasts after stimulation with IL-1*β*, and in gingival epithelial cells after stimulation with TNF-*α* [17, 21, 22]. These studies suggest a role of HMGB1 in periodontal diseases. However, whether periodontal ligament fibroblasts express and release HMGB1 in an inflammatory environment has not yet been examined. Also, the profile of HMGB1 expression during the initiation and progression of experimental periodontal disease was not evaluated yet. Thus, the aim of this study was to investigate *in vitro* the expression profile of HMGB1 in mouse periodontal ligament fibroblasts (mPDL) stimulated with *Escherichia coli* LPS or IL-1*β*, mimicking the inflammatory condition present in periodontitis. In addition, this study evaluates *in vivo* the expression of HMGB1 during the initiation and progression of experimental periodontal disease induced in two rat models.

## 2. Materials and Methods

### 2.1. Experimental Design

#### 2.1.1. *In Vitro* Study

*Cell Culture*. Immortalized mPDL cells were kindly provided by Professor Dr. Martha Somerman (National Institutes of Health). mPDLs were kept in DMEM culture medium (Invitrogen, Carlsbad, CA) supplemented with 10% FBS (Invitrogen), 100 U/mL penicillin, and 100 *μ*g/mL streptomycin at 37°C in a humidified atmosphere of 5% CO_2_. FBS was reduced to 0.03% twelve hours prior to stimulus induction.

*Cell Stimulation with LPS and IL-1*β**. mPDL cells were cultured in 6-well plates (3 × 10^5^ cells/well) and stimulated with either 1 *μ*g/mL *Escherichia coli* LPS (Sigma-Aldrich, St. Louis, MO) or with 5 ng/mL IL-1*β* (R&D Systems, Minneapolis, MN) for 4, 8, 12, and 24 h. The control group was left unstimulated and all experiments were performed in duplicate.

#### 2.1.2. *In Vivo* Study

The animal study protocol was approved by the local Ethical Committee for Animal Experimentation and conducted according to the ARRIVE guidelines. Thirty-six male adult Wistar rats, obtained from the Multidisciplinary Center for Biological Investigation on Laboratory Animal Science (CEMIB-UNICAMP), Campinas, Brazil, with average weight of 250 g, were randomly divided into three groups: a negative control (sham-operated) group and two different experimental groups in which one of two experimental periodontal disease models were used: *Escherichia coli* LPS- (Strain 055:B5, Sigma-Aldrich) or ligature-induced periodontal disease, in which cotton threads were placed around the cervical area of both lower first molars after general anesthesia (ketamine chlorhydrate: 0.08 mL/100 g body weight and xylazine chlorhydrate: 0.04 mL/100 g body weight, administered via intramuscular injection). For the injections procedures, 3 *μ*L of a 20 *μ*g/*μ*L *Escherichia coli* LPS was delivered into the palatal gingivae between both maxillary first and second molars 3 times per week. The injections of LPS occurred after sedation of the animals with isoflurane (Baxter Healthcare Corp., Deerfield, IL). These injections were performed using a custom designed 0.375 in × 33 gauge needles, attached to a 10 *μ*L Hamilton syringe (Hamilton Company, Reno, NV). After 7, 15, and 30 d of the periodontal disease induction, 3 animals from the control group and 9 animals from the experimental groups were euthanized per time period. Mandibulae and maxillae hemisections were submitted for histological processing for stereometry and immunohistochemistry analyses. The other half was used for qPCR and Western blot analyses from the gingival tissues and for macroscopic assessment of bone levels.

### 2.2. Real-Time PCR

Total RNA was extracted from mPDL cells using trizol reagent, according to the manufacturer's protocol (Invitrogen) and from rat gingival tissue samples using *RNAqueous-4PCR* kit (Ambion, Applied Biosystems, Foster City, CA). The quantity and purity of total RNA were determined on a NanoVue UV/Visible Spectrophotometer (GE Healthcare, Piscataway, NJ). Complementary DNA was synthesized by reverse transcription of 500 ng and 400 ng of total RNA from the cells and gingival tissue samples, respectively, following the manufacturer's protocol (TaqMan Reverse Transcription Reagents Kit, Applied Biosystems). Quantitative PCR was performed using a Step One thermocycler (Applied Biosystems). The target gene, assay ID number, accession number of the reporter probe, and amplicon were (1) rat GAPDH ID Rn99999916_s1, accession NM_017008.3, and 87 bp; (2) rat HMGB1, ID Rn00820665_g1, accession NM_012963.2, and 70 bp; (3) mouse GAPDH, ID Mm03302249_g1, accession NM_032110.1, and 70 bp; and (4) mouse HMGB1, ID Mm00849805_gh, accession NM_034569.1, and 158 bp. The cycling conditions used for all primers were preoptimized: 50°C for 2 min and 40 cycles of 95°C for 15 seconds and 60°C for 1 min. Determination of the relative levels of gene expression was performed using the cycle threshold (CT) method in reference to housekeeping gene GAPDH for the *in vivo* or beta-actin for the *in vitro* study, as they did not alter by the experimental conditions.

### 2.3. Western Blot

Total protein was extracted from gingival tissues for the *in vivo* study and from conditioned medium for the *in vitro* study. For the *in vitro* study, conditioned medium was collected and concentrated with centrifuge filter devices (Microcon YM-10, Millipore Corp.) at 14,000 ×g for 25 min at room temperature (RT). For the *in vivo* study, total proteins were extracted from gingival tissue samples using a detergent-based extraction buffer (T-PER, Tissue Protein Extraction Reagent, Pierce, Rockford, IL) containing a protease inhibitor cocktail (Protein Stabilizing Cocktail, Santa Cruz Biotechnology, Santa Cruz, CA). Tissue samples were macerated in 30 *μ*L buffer and centrifuged for 5 min at 13,000 ×g RPM at 4°C. The concentrated supernatants and the total protein from gingival tissues were quantified using a Bradford-based assay (RC-DC assay, Bio-Rad Laboratories Inc., Richmond, CA). Thirty *μ*g of total protein was added to a sample buffer consisting of 2% SDS, 10 mM of DTT, glycerol, and bromophenol blue dye (Cell Signaling Technology, Danvers, MA), heated and denatured at 97°C for 5 min and chilled on ice for 5 min before loading on 10% SDS-polyacrylamide gels. Electrophoresis on discontinuous acrylamide gels was carried out at constant 100 V for 90 min and subsequently electrotransferred to 0.4 *μ*m nitrocellulose membranes using a 300 mA constant current for 1 h. The membranes were blocked for 1 h in Tris-buffered saline containing 5% nonfat dry milk and 0.1% Tween-20 and subsequently washed for 5 min (three times) with TBS-0.1% Tween-20 (TBS-T). The membranes were then incubated with rabbit polyclonal primary antibody (1 : 500, ab18256, Abcam, Cambridge, MA) overnight at 4°C. The secondary antibodies conjugated to horseradish peroxidase (1 : 1000, anti-rabbit, Santa Cruz Biotechnology) were incubated for 1 h at RT. Data from the *in vivo* study were normalized to levels of GAPDH.

### 2.4. Stereometric Analysis

Tissue blocks were fixed in 4% buffered formalin for 48 h, decalcified in EDTA (0.5 M, pH 8.0) for 3 months at RT, and embedded in paraffin. Semiserial 5 *μ*m sections were obtained in the frontal plane (buccal-lingual orientation) and stained with hematoxylin and eosin. Three different sections spaced 300 mm apart, representing the mesial, mid, and distal areas of the teeth, were examined from each rat. The total number of rats used for each group was six. Images were captured using a digital camera (Leica DFC 300 FX) on an optical microscope (Diastar-Cambridge Instruments) set at 20x magnification. For the measurement, a lower limit was represented by a horizontal line drawn at the top of the bone crest, and the soft tissue above this line was divided into the following thirds: (1) near to the tooth, (2) the middle portion, and (3) near the oral epithelium (Figure 2). Inflammatory parameters were determined by scores [23]. The inflammatory scores were 0, no inflammatory cells; 1, slight inflammation (a few inflammatory cells); 2, moderate inflammation (remarkable inflammatory cells scattered throughout the connective tissue above the bone crest); and 3, severe inflammation (predominance of inflammatory cells). The analysis was conducted by a single, calibrated examiner (JACS) that was masked to the experimental groups. Scores for each group (control, LPS, or ligature) and area (mesial, mid, or distal) were averaged for each period and used to determine inflammation severity.

### 2.5. Immunohistochemical Analysis

Immunohistochemical analysis for HMGB1 protein within the periodontal tissues was performed by the avidin-biotin peroxidase (ABC) method using the LSAB kit (Dako, Glostrup, Denmark) according to manufacturer's instructions. Tissue sections with 5 *μ*m thickness mounted on silanized slides (Dako) were used. Antigen retrieval was carried out by hitting cuts in the Rodent Decloaker reagent (Biocare Medical; Concord, CA) in a Decloaking Chamber (Biocare Medical) for 30 min at 80°C. Endogenous peroxidase activity was blocked with a 3% hydrogen peroxide solution and nonspecific binding blocking was performed in 3% bovine serum albumin (BSA, Sigma) in PBS. As a negative control, primary antibody was omitted and the sections were incubated with 1% PBS to assess background staining. Sample sections were incubated overnight with a rabbit polyclonal primary antibody (1 : 200, ab18256, Abcam). Slides were stained with DAB (Dako) and counterstained with Carazzi's hematoxylin. One blinded and calibrated examiner to the experimental groups (RSM) analyzed a total of 4 digital images per group/time point in an optical microscopy (LEICA microsystem GmbH, Wetzlar, Germany) at 40x magnification. Positive cells were counted in a rectangular area of interest (AOI) measuring 32,400 *μ*m^2^, with the apical border of junctional epithelium used as the coronal limit and the tooth root as the medial limit of the AOI. The identification of the cells was made by a pathologist who had evaluated the immunohistochemical images. The cells pointed with black arrows on Figure 3 were considered fibroblasts due to their morphology (elongated nuclei) and to their localization (accompanying collagen fibers of the periodontal ligament).

### 2.6. Macroscopic Alveolar Bone Evaluation

Pieces were removed from 70% alcohol, dried, immersed for 5 min in a solution containing 0.7 g/L of methylene blue, and washed with tap water to remove dye excess. Alveolar bone resorption was measured on the images in 3 different regions for the lower first molar: mesial, middle, and distal roots. For the maxilla, 3 different regions were also measured: middle and distal roots of the first molar and mesial root of the second molar. Linear measurement of alveolar bone loss was macroscopically performed at the lingual surface of the upper and lower first molars by staining the hemimaxillas with methylene blue. Digital images of the stained surfaces were obtained at a standardized 90° angle with the midlingual aspect of the teeth with a stereomicroscope (Leica MZ6—20x magnifications). The measurement was performed by an examiner (ESMP) masked to the experimental groups using Image tool 3.0 software. Alveolar bone resorption comprised the linear vertical distance from cement-enamel junction (CEJ) to bone crest surfaces. An increase on the measurement of exposed roots in comparison to control teeth indicated alveolar bone loss.

### 2.7. Statistical Analysis

For both studies, in each experimental period, the results were compared to the control group using unpaired Student's *t*-test. One-way analysis of variance test [3] followed by the Tukey post hoc test was used to evaluate significant intergroup differences. Significance level was considered when *P* < 0.05. Statistical analyses were performed using GraphPad Prism 5 software (GraphPad Inc., San Diego, CA).

## 3. Results

### 3.1. HMGB1 Is Expressed and Released by mPDL Cells after LPS or IL-1*β* Stimulus

**Figure 1:**
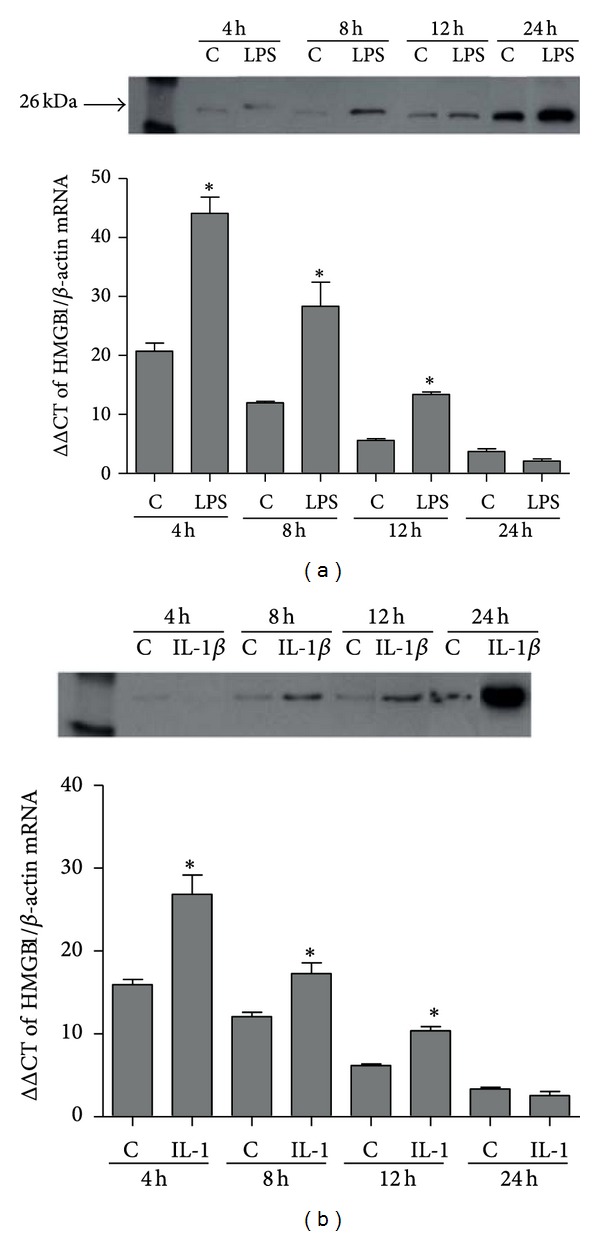
HMGB1 protein release in supernatants from murine periodontal ligament fibroblasts (mPDL) assessed by Western blot after stimulation with LPS (*Escherichia coli*) (a) or IL-1*β* (b) for 4, 8, 12, and 24 hours. HMGB1 mRNA expression assessed by qPCR in MPDL cells after stimulation with LPS or IL-1*β* for 4, 8, 12, and 24 hours. C means control group. *Significant difference compared to control (*P* < 0.05).

mPDL cells were demonstrated to constitutively express HMGB1. The qPCR data demonstrated a significant increase (*P* < 0.05) in the HMGB1 mRNA expression in the cells stimulated with both LPS and IL-1*β* compared to the control group after 4, 8, and 12 h. For protein analysis by Western blot, HMGB1 was shown to be released by mPDL cells. Stimulation with LPS or IL-1*β* induced higher HMGB1 protein production and extracellular release compared to the control group in a time-dependent manner (Figures 1(a) and 1(b)).

### 3.2. Severity of Inflammation in Gingival Tissues during Experimental Periodontal Disease

**Figure 2:**
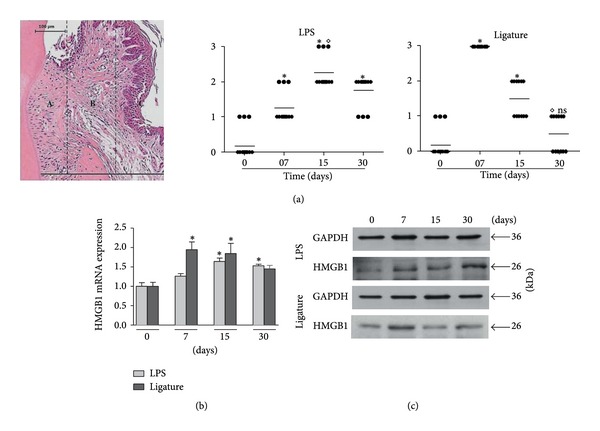
(a) Stereometric analysis of the inflammatory cells. Representative histologic section shows the 3 areas used for analysis. Graphs show average ± standard deviation of scores obtained in the LPS- and ligature-induced periodontal disease models. The LPS model is associated with a sustained inflammatory reaction, while the ligature model presented higher density of cellular infiltrate at 7 days and a decrease on the severity of inflammation at 30 days. (b) and (c) HMGB1 mRNA and protein expression assessed by qPCR (b) and Western blot (c) during the course of LPS- and ligature-induced periodontal disease. *Significant difference compared to control (*P* < 0.05).

The LPS-PD model showed a sustained density of inflammatory cells in all experimental periods with significant difference (*P* < 0.05) compared to control. At 15 d, the inflammatory response was slightly higher with statistical difference (*P* < 0.05) only in relation to 7 d. For the ligature model, the peak of inflammation occurred at 7 d following disease induction, with a subsequent decrease in the inflammatory response in a time-dependent manner (Figure 2(a)).

### 3.3. HMGB1 Is Expressed in Gingival Tissues during Experimental Periodontal Disease

**Figure 3:**
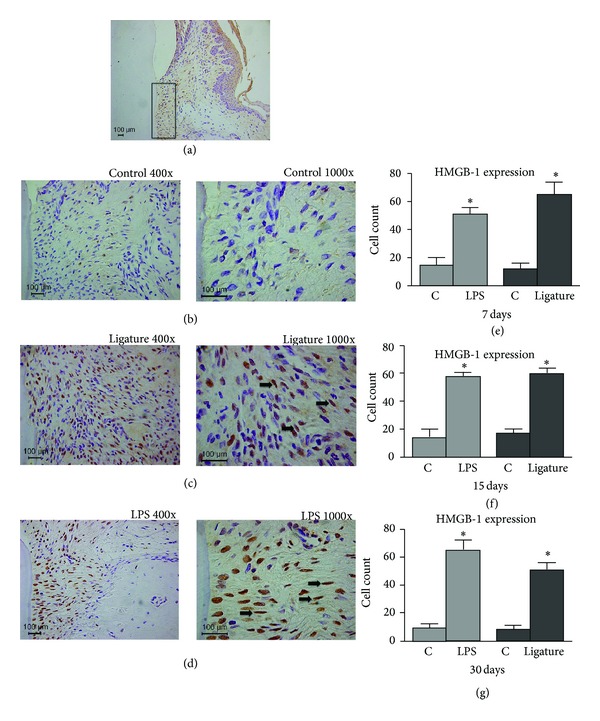
Immunohistochemical analysis of the inflammatory process. (a) Representative lower magnification of the images (b) to (d) showing the selected region in the interproximal area of the first maxillary and mandibular molars. (b) Images of the control group showing few stained cells at 7 days. (c) and (d) Images of the ligature and LPS groups, respectively, showing many stained cells at 7 days. (e) to (g) Graphs with mean ± standard deviation of the number of positive cells to HMGB1 at 7, 15, and 30 days. *Significant differences compared to control (*P* < 0.05).

A significant increase (*P* < 0.05) was observed in the HMGB1 mRNA expression for both *in vivo* experimental periodontal disease models (Figure 2(b)). Compared to the control group, gingival tissues stimulated with LPS yielded higher HMGB1 expression at all time points, especially at 15 d and 30 d after disease induction. Tissues from the ligature model presented higher expression of HMGB1 at 7 d and 15 d, followed by a decrease at 30 d. Also, HMGB1 protein production was confirmed in gingival tissues by Western blot analysis, mainly at 30 d for LPS-PD and 7 d for ligature model (Figure 2(c)). Immunohistochemical analysis revealed a significant increase in the number of HMGB1-positive cells in both experimental groups compared to the control group that was poorly stained (*P* < 0.05). HMGB1 was more expressed in the nucleus and cytoplasm of the cells than in the extracellular space. The increase in HMGB1-positive cells persisted during all of the experimental periods but without statistical significance between the LPS-PD and ligature groups (Figures 3(a) to 3(g)).

### 3.4. Alveolar Bone Loss during LPS- and Ligature-Induced Periodontal Disease

**Figure 4:**
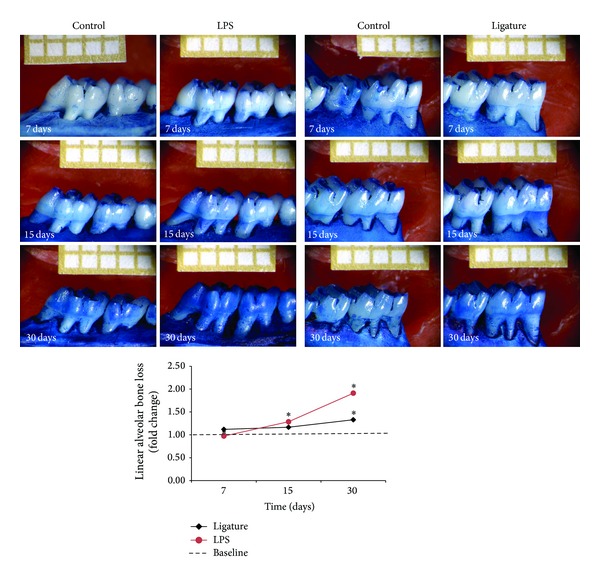
Linear alveolar bone loss was measured macroscopically in the lingual surface of the upper and lower first molars corresponding to LPS (left) and ligature (right) models, respectively. Representative samples stained with methylene blue for each group and period are shown. Graph with average ± standard deviation of linear alveolar bone loss in fold change. *Significant difference among experimental periods (*P* < 0.05).

Different patterns of bone loss could be detected in both experimental periodontal disease models (Figure 4). For the LPS model, alveolar bone loss occurred as early as 7 d, followed by a significant increase at 15 d and 30 d. The groups presented a significant difference among experimental periods (*P* < 0.05). In the ligature model, alveolar bone loss was also detected at 7 d with significant increase in bone loss only at 30 d after disease induction.

## 4. Discussion

Experimental models simulating periodontal diseases are widely used for a better understanding of their pathogenesis, focusing on the molecular mechanisms and mediators involved in these processes [24, 25]. The purpose of using two different models of periodontal disease was to compare the HMGB1 expression in the ligature model which has the participation of live microorganisms present in the dental biofilm accumulated and induction of a more complex host response to the LPS model which induces chronic inflammation and host response specifically through TLR4 activation [24]. The *in vitro* model demonstrated that HMGB1 is expressed and released by PDL fibroblasts upon inflammatory signals, demonstrating the participation of HMGB1 in response to putative inflammatory signals from periodontitis. This response is in accordance with the findings from other cell types that showed that HMGB1 is released after inflammatory stimuli [17, 21, 22].

Several studies have used these same immortalized mPDL cells and obtained reliable results [26, 27]. The immortalization is performed after isolation of the cells in order to maintain cell phenotypes and promote cell proliferation. In addition, these immortalized cells in culture maintain gene expression profiles seen in primary cultures and *in situ* [28].

Consistent with the *in vitro* findings, the *in vivo* data demonstrated an increase in HMGB1, at both mRNA and protein levels, within gingival tissues afflicted with experimental periodontal disease. In the ligature model, HMGB1 expression could be compared to the expression profile of TNF-*α* and IL-6 from our previous study in which higher expression of these genes occurred at days 7 and 15 followed by a reduction at 30 days [29]. These *in vivo* results corroborate also with a clinical study that have demonstrated high levels of HMGB1 in gingival tissues and gingival crevicular fluid of chronic periodontitis patients compared to control [30]. In addition, this clinical study showed that HMGB1 responds in a similar way to other cytokines during disease progression. Thus, HMGB1 is being expressed similarly and concomitantly with other cytokines during periodontal disease induction, suggesting a role of this molecule in the inflammatory events occurring in periodontal disease initiation and progression.

The increased expression of HMGB1 in inflamed tissues at 7 and 15 days for ligature and LPS-PD models, respectively, followed by decreased expression at 30 days, is strongly associated with the inflammatory condition. These findings were confirmed by stereometric analysis suggesting that inflammatory cells, residing cells, and/or their cytokines present in gingival tissues activate the production and release of HMGB1 during periodontal disease induction. In addition, HMGB1 protein was detected by Western blot analysis at all time points for both periodontal disease models.

Immunohistochemical staining revealed that HMGB1 expression was increased during the course of periodontal disease in both models and was maintained from 7 d through 30 d. The continued expression of HMGB1 over time occurs in conjunction with the maintenance of stimuli, thus acting as an important signal for progressive periodontal destruction [21]. These results suggest that periodontal disease could contribute to HMGB1 translocation from the nucleus to the cytoplasm in the epithelial and connective cells. Our results are in agreement with a previous study that also detected more HMGB1 expression intranuclearly and cytoplasmically than extracellularly in the presence of a chronic inflammatory disease [31]. In addition, another study [21] also observed that most of the HMGB1-positive expression was in the nucleus and cytoplasm of epithelial cells, although our study has evaluated the HMGB1 within the connective tissue. Unlike the findings of other studies [21, 32] with regard to the localization of HMGB1, our results showed that although the identification of the cells were preliminary, immunohistochemical analysis suggests that residing cells of the periodontal ligament express more HMGB1 in the experimental groups compared to control groups. This fact could be explained by the methodology employed in this study, where the periodontal disease was experimentally induced in rats, compared to human patients with naturally occurring chronic periodontal disease.

Although identifying which cells are expressing HMGB1 in the gingival tissue samples was not our purpose, Kalinina and collaborators [33] have demonstrated in atherosclerotic lesions that several cells such as endothelial cells, smooth muscle cells, and macrophages were producing HMGB1, suggesting that different cell types in the periodontal lesions can also be expressing HMGB1.

Previous studies have suggested a participation of HMGB1 in the osteoclastogenesis process [7, 34]. In the present study, the alveolar bone loss increased during the progression of experimental periodontitis, showing the efficiency of these PD models. Using the LPS-PD model, significant differences were found among all experimental time periods (*P* < 0.05), with the greatest bone loss occurring at day 30. For the ligature model, a significant degree of bone loss occurred at day 30 (*P* < 0.05). The elevated HMGB1 expression and release may have contributed together with other cytokines for the alveolar bone loss observed. Also, continuous release of HMGB1 over time, as shown by Western blot, could amplify the signals for periodontal destruction and act in synergism with other cytokines such as IL-1*β* and TNF*α*.

Zhou and collaborators [7] have shown *in vitro* that RANKL promotes HMGB1 translocation and release from macrophages. Also, RANKL seems to signal extracellular HMGB1 to induce osteoclastogenesis and activate osteoclasts. *In vivo *studies have shown similar results indicating that, in the presence of extracellular HMGB1, RANKL is able to induce more evident osteoclastogenesis [7]. *In vitro* and *in vivo* studies have shown that HMGB1 may be essential to the differentiation of osteoclast precursors by RANKL [35]. Also, HMGB1 was shown to be associated with RANKL in the regulation of host response during periodontal repair following orthodontic tooth movement in rats [36]. More studies are necessary to investigate the role of HMGB1 in osteoclastogenesis during periodontal disease to better elucidate whether this protein has important participation, whether alone or in cooperation with other osteoclastogenesis-inducing signaling molecules.

Another interesting observation about HMGB1 is that it can bind to IL-1*β* in cells stimulated with this cytokine, meaning that the association of these molecules enhances the proinflammatory properties of IL-1*β*, like bone loss activation [37]. This demonstrates that even if HMGB1 itself has minimal proinflammatory characteristics, it is able to amplify the response of other molecule [22].

Other inflammatory stimuli such as mechanical loading or thermal stimulus, considered as sterile inflammatory/necrosis stimuli, have shown to express and release HMGB1 in human PDL cells [38, 39]. Furthermore, increased macrophage migration and differentiation into osteoclast were observed in macrophages cultured with conditioned medium of those PDL cells subjected to mechanical loading or thermal stimulus due to the presence of HMGB1 in PDL cells. This evidence suggests that HMGB1 has a role not only as a proinflammatory cytokine but also as a proliferative signal in periodontal repair.

Lately, contradictory results regarding HMGB1 activity in stimulating or noninflammation have emerged. Studies found that the redox state of HMGB1 is the main fact determining its immune activity [40–44]. These authors suggest that modification of the oxidative environment can decrease inflammation and tissue damage by modulating HMGB1 functions. Thus, different forms of HMGB1 based on the redox status exist. HMGB1 can induce cytokine production or not and even tissue repair depending on its structure.

In the *in vitro* study, *E. coli* LPS was used. Although it has some advantages such as being a strong TLR4 agonist and having great potential to induce proinflammatory cytokines in PDL cells [45, 46], *E. coli* LPS has some limitations like the fact that it is not found in the oral environment. As a result, more studies evaluating the effect of heat-killed bacteria or LPS from periodontopathogens are necessary to deeply investigate the role of HMGB1 in periodontal disease.

## 5. Conclusions

Our *in vitro* and *in vivo* studies have demonstrated, respectively, that mDPL fibroblasts are able to synthesize HMGB1 and that gingival tissues afflicted with experimental periodontal disease also express HMGB1, indicating that this protein may be associated with the onset and progression of periodontal disease. Based on these results, further functional studies are necessary to interpret the role of HMGB1 participation in chronic inflammatory diseases. Also, it is suggested that additional investigations evaluating the mechanisms of HMGB1 participation on periodontal tissue destruction in periodontal disease patients appear warranted.
